# Nosocomial meningitis diagnostic test characteristics: a systematic review

**DOI:** 10.1016/j.infpip.2024.100402

**Published:** 2024-09-23

**Authors:** David Granton, Joseph Brown, Shannon M. Fernando, Dipayan Chaudhuri, Isaac I. Bogoch, Christine Soong, Marina Englesakis, Bram Rochwerg, Eddy Fan

**Affiliations:** a Department of Medicine, University of Toronto, Toronto, ON, Canada; b Michigan State University College of Human Medicine, Michigan, USA; c Department of Critical Care, Lakeridge Health Corporation, Oshawa, ON, Canada; d Clinical Epidemiology Program, Ottawa Hospital Research Institute, Ottawa, ON, Canada; e Department of Medicine, McMaster University, Hamilton, ON, Canada; f Divisions of Internal Medicine and Infectious Diseases, University Health Network, Toronto, ON, Canada; g Division of General Internal Medicine, Sinai Health, University of Toronto, Toronto, ON, Canada; h Institute of Health Policy Management and Evaluation, University of Toronto, Toronto, ON, Canada; i Library & Information Services, University Health Network, Toronto, ON, Canada; j Department of Health Research Methods, Evidence and Impact, McMaster University, Hamilton, ON, Canada; k Interdepartmental Division of Critical Care Medicine, University of Toronto, Toronto, Canada

**Keywords:** Nosocomial, Meningitis, Lumbar puncture

## Abstract

**Background:**

The incidence of nosocomial meningitis, and utility of lumbar puncture, is unclear in hospitalized patients without preceding neurosurgery or head trauma.

**Aim:**

We planned for a systematic review and meta-analysis to evaluate accuracy of clinical features and diagnostic utility of lumbar puncture in nosocomial meningitis.

**Methods:**

We searched MEDLINE, MEDLINE In-Process/ePubs, EMBASE, Cochrane Central Register of Controlled Trials, Cochrane Database of Systematic Reviews, and Web of Science from inception until June 5, 2024. We included studies evaluating utility of clinical features, or lumbar puncture, to rule out nosocomial meningitis in patients without preceding neurosurgery or head trauma. We excluded studies examining community acquired meningitis, outbreaks, HIV positive individuals, and case reports. Outcomes included incidence, risk factors and diagnostic accuracy of clinical features for nosocomial meningitis, and lumbar puncture complications. Given few included studies and heterogeneity, we could only summarize incidence of nosocomial meningitis.

**Findings:**

Of 13,302 citations, we reviewed 197 manuscripts and included 6. There were 23 of 333 (6.9%, very low certainty) positive lumbar punctures among individuals who underwent lumbar puncture to rule out nosocomial meningitis.

**Conclusions:**

There were insufficient data to evaluate the diagnostic accuracy of lumbar puncture in nosocomial meningitis in patients without preceding neurosurgery or head trauma. Very low certainty evidence indicates the incidence of nosocomial meningitis is low in this population. Given complications and costs associated with lumbar puncture, future studies should evaluate its utility in nosocomial meningitis. In the meantime, it may be reasonable to reserve lumbar puncture to instances of high suspicion.

## Introduction

Nosocomial meningitis is associated with high morbidity and mortality and is distinct from community-acquired meningitis in terms of risk factors and microbiology. Common risk factors for nosocomial meningitis include preceding neurosurgery, central nervous system instrumentation, and complicated head trauma, [[1], [2], [3], [4], [5]] however nosocomial meningitis may still occur in adults without these underlying risk factors [[2], [3], [4],^6^].

Alterations in level of consciousness and fever are common in hospitalized patients [[7], [8], [9], [10]]. The incidence of fever in hospitalized patients varies widely, and has been estimated to occur in 2–31% of inpatients [10,11], while the incidence of delirium was as high as 81% in one study evaluating adults admitted to ICU [8]. When this combination of symptoms arise in hospitalized patients at our centers of practice, clinicians and trainees sometimes consider nosocomial meningitis on their differential diagnosis. This prompts consideration of a lumbar puncture (LP), however its use in this clinical setting is uncertain. To date, most studies examining nosocomial meningitis have focused on individuals with preceding neurosurgery, indwelling intracranial devices, or head trauma. The 2017 Infectious Diseases Society of America guidelines on health-care associated meningitis and ventriculitis focus solely on this population. Their recommendations surrounding diagnosis feature (among other factors): a) clinical signs such as new headache, fever, altered mental status, erythema surrounding indwelling devices b) cerebrospinal fluid (CSF) studies such as positive cultures, pleocytosis, hypoglycorrhachia, elevated protein, and lactate c) neuroimaging. They do not provide guidance on nosocomial meningitis in the absence of preceding neurosurgery, indwelling intracranial devices, or head trauma [12]. The incidence of nosocomial meningitis in hospitalized patients without these risk factors is unclear. Additionally, it is unclear if the clinical features that increase the likelihood of a diagnosis of nosocomial meningitis in non-neurosurgical and non-head trauma patients are similar to either those described in community acquired meningitis [13,14] or meningitis in the setting of preceding neurosurgery, indwelling intracranial devices, or head trauma [1,12]. The studies that are available in this population are imprecise, and generally report a low incidence of nosocomial meningitis [[15], [16], [17]]. Given the unclear incidence, and lack of described predictive clinical features, it remains unclear if there is utility in performing an LP to rule out meningitis in this population.

The objective of this systematic review was to summarize the accuracy of clinical signs and symptoms in the diagnosis of nosocomial meningitis in hospitalized patients without preceding neurosurgical intervention or head trauma and examine the diagnostic utility of LP in this population.

## Materials and methods

This systematic review was conducted in accordance with the Preferred Reporting Items for Systematic Reviews and Meta-Analyses (PRISMA) recommendations [18] and was pre-registered on PROSPERO (CRD42021274321).

### Data sources and searches

We performed a systematic search using six databases (MEDLINE, MEDLINE In-Process/ePubs, EMBASE, Cochrane Central Register of Controlled Trials, Cochrane Database of Systematic Reviews and Web of Science) from inception until June 5, 2024 (Supplementary Material: Initial Search Strategy). We searched for studies of hospitalized adults with suspected nosocomial meningitis using keywords “meningitis” together with “nosocomial” or “iatrogenic” or “hospitalized” or “health-care associated”. We did not exclude studies based on quality, language or if published exclusively in abstract form. We also screened citations of included studies for any potentially additional studies (Supplementary Material: Initial Search Strategy).

### Study selection

Two reviewers (DG, JB) screened citations independently and in duplicate. We initially reviewed citations by title and abstract, and then for any identified as potentially relevant by either reviewer, by full text. Disagreements at full text review were resolved by third party adjudication (EF). We recorded reasons for trial exclusion during full text screening only. Studies that were not published in English were translated using Google Translate [19].

We included studies featuring adult inpatients who developed clinical features (fever, altered mental status etc.) prompting a LP to rule out nosocomial meningitis. We included studies where LP was used as the reference standard for diagnosis of meningitis. We included studies even if they lacked reporting of individual clinical features, as they would still give information on the incidence of nosocomial meningitis and utility of LP. We excluded case reports, however all other study designs were eligible for inclusion. We excluded studies that exclusively studied patients with preceding neurosurgery, history of trauma, and previous or current indwelling intracranial devices. We excluded these patients given these are well established risk factors for nosocomial meningitis. In patients with preceding neurosurgery and clinical presentation compatible with meningitis, the incidence has been described as high as 42% [1,[20], [21], [22], [23], [24], [25], [26], [27]]. We also excluded studies that exclusively featured patients in which LP was performed to rule out community-acquired meningitis, or patients who initially presented to hospital with symptoms possibly suggestive of meningitis such as fever, headache or altered level of consciousness. These studies that featured mixed populations were only included if they specifically reported on outcomes in a subgroup of patients that met the eligibility criteria of this review. We excluded case series that reported only on confirmed positive cases of nosocomial meningitis, as these would not give data on the incidence of nosocomial meningitis or the diagnostic utility of clinical features. We excluded studies that reported patients diagnosed with HIV only. We also excluded any studies that examined meningitis outbreaks or examined a single microbiological isolate given that these typically describe confirmed positive cases, and also would not be representative of the true incidence of nosocomial meningitis given the nature of an outbreak.

We included the following outcomes of interest: the incidence of nosocomial meningitis in hospitalized patients undergoing LP, accuracy of clinical features for diagnosis of nosocomial meningitis, LP related complications as well as clinical risk factors for nosocomial meningitis.

### Data extraction

Two reviewers (DG, JB) performed data abstraction independently and in duplicate using a standardized data abstraction form. We resolved disagreements in data abstraction with consensus and third-party adjudication, if required (EF). We abstracted data including study details (location, objective, funding etc.), inclusion and exclusion criteria, minimum duration of hospital and/or ICU length of stay prior to LP, mean time from hospital and/or ICU admission to LP, proportion of patients receiving antibiotics prior to LP, definition of nosocomial infection, meningitis diagnostic criteria, number of patients included and outcomes. We considered a “positive” LP to be one which met criteria for a case of nosocomial meningitis, as defined by study authors.

### Data analysis

We initially developed and planned for a quantitative meta-analysis evaluating our outcomes of interest. However, the reporting of LP related complications and risk factors for meningitis was infrequent among included studies. Additionally, there was clinical and methodological heterogeneity and often lack of reporting of clinical features prompting LP across studies. These factors, along with the small number of included patients precluded pooling for these outcomes. Instead, we narratively described these outcomes. We summarized the incidence of nosocomial meningitis across studies using descriptive statistics. We narratively described differences between study characteristics including differences in minimum and mean ICU or hospital length of stay prior to LP, definition of nosocomial acquisition, meningitis diagnostic criteria and proportion of patients receiving antibiotics prior to LP (Table I: Characteristics of Included Studies). We were also unable to perform any of the pre-defined subgroup analyses which included comparing: immunocompromised vs. immunocompetent patients, ICU vs. ward patients, high vs. low risk of bias (ROB) studies, minimum length of stay prior to LP (over one week vs. under one week) and indication for LP. We have included the initial statistical analysis plan in the supplement (Supplementary Material: Statistical Analysis Plan).

**Table I:** Characteristics of included studies

Study (Author, Year)	Multi/Single site and location	Population	Age (Mean± SD in years)	Minimum duration of hospital length of stay to LP	Mean duration from ICU admission to LP	N(%) of patients received antimicrobials before LP	ICU or ward patients	Definition of nosocomial case	Meningitis/LP diagnostic criteria	Number of LPs performed	Number of LPs meeting inclusion criteria	Number of positive LPs
Jackson, 2006 [15]	Single site-Washington, DC, USA	**Inclusion:** All MICU patients that underwent LP during stay **Exclusion:** Patients with indwelling CNS devices	57.3± 20.0	All >48 hours	**Positive LP:** 13.4± 20.5 hours **Negative LP:** 103.4± 162.9 hours	25 (80.7%)	All ICU	Occurred > 48 hours after hospital admission (1988 CDC definitions also used)	Compatible clinical presentation with either >10 leukocytes/mm^3^ neutrophilic predominance, or positive CSF gram stain or culture, or both	69	31	5 (16.1%)^a^
Metersky, 1997 [16]	Multisite- Hartford and Farmington, CT, USA	**Inclusion:** All nonsurgical patients over the age of 15 years with LP performed to investigate acute CNS infection. **Exclusion:** Patients with previous CNS procedures, suspected neurosyphilis or Lyme disease, previously diagnosed infection	59± 20	All > 48 hours	N/A	N/A	N/A	Indications for LP arose > 48 hours following admission	i) Bacterial- proven by positive culture or appropriate stain^b^ ii) Aseptic- typical clinical presentation with associated CSF lymphocytic pleocytosis and no pathogen identified	232	51	0
Warshaw, 1993 [17]	Single site- Cincinnati, OH, USA	**Inclusion:** Adults 65 years or older that received LP and CSF analysis to evaluate fever and mental status changes **Exclusion:** Patients with LP performed for other indications, or patients with delirium that did not have LP performed	76± 7.6^c^	N/A	N/A	N/A	Medical Ward and ICU	N/A	N/A	81	24	0
Adelson-Mitty, 1997 [30]	Single site- Boston, MA, USA.	**Inclusion:** All SICU patients with LP performed **Exclusion:** Recent neurosurgical procedure, indwelling intracranial pressure monitor or ventriculostomy, recent CNS injury, LP performed outside SICU	61 (Range: 16–96)	N/A	12± 15 days	53 (75%)	All ICU	N/A	CSF white blood cell count >10 cells/ml and positive gram stand and/or culture	204	70	0
Khasawneh, 2015 [31]	Single site- Amarillo, TX, USA	**Inclusion:** All patients admitted to MICU who had an LP performed **Exclusion:** Patients admitted to ICU service other than MICU, LP performed before admission to MICU, LP performed to rule out diagnosis other than meningitis, CSF collected via intracranial or lumbar drain	**Positive LPs**^c^**:** 54.5± 16.1 **Negative LPs**^c^**:** 54.3± 16.2	All > 48 hours	2.8 days^c^	117 (73.8%)^c^	All ICU	Diagnosis made > 48 hours after admission (1988 CDC definitions also used)	Either positive CSF microbial stain or culture, or CSF pleocytosis (cell count >5)	168	51	10 (19.6%)
van Zeggeren, 2023 [32]	Multisite - Netherlands	**Inclusion:** Adults with suspected non-surgical nosocomial CNS infection **Exclusion:** Recent neurosurgery or TBI (within 1 month), neurological device in situ	Median: 63 (IQR: 50–70)	All >48 hours (unless within 1 week after discharge)	N/A	88 (79%)	Medical ward, emergency department, and ICU	Suspicion of CNS infection occurring > 48 hours after admission or within 1 week after hospital discharge	Positive gram stain, culture, or polymerase chain reaction of the CSF or in blood in combination with CSF leucocytosis. If not present, diagnosis could be made “based on the investigators' classification”	1275	114	16^d^

CSF – Cerebrospinal Fluid, CDC – Centers for Disease Control and Prevention, CNS – Central Nervous System, LP – Lumbar Puncture, MICU – Medical Intensive Care Unit, TBI – Traumatic Brain Injury, SICU – Surgical Intensive Care Unit.

a Of the 5 positive LPs, 2 were cryptococcal meningitis in patients with HIV, 1 was tuberculosis meningitis, 1 was previously diagnosed pneumococcal bacteraemia, 1 was previously diagnosed viral encephalitis.

b One case defined as bacterial meningitis, despite a negative culture and gram stain, because of neutrophilic pleocytosis and a typical clinical presentation and response to therapy.

c Includes community acquired and nosocomial meningitis cases.

d Of the 16 positive LPs, 4 were bacterial (including 1 pneumococcal meningitis in a patient recently discharged), 9 were viral, 2 were fungal and 1 was parasitic.

### Quality and certainty assessment

We initially planned to use the Quality Assessment of Diagnostic Accuracy Studies (QUADAS-2) to evaluate individual study ROB and applicability concerns [28] related to diagnostic test accuracy of clinical signs and symptoms. However, we were unable to pool the diagnostic performance of clinical features in nosocomial meningitis due to the small number of patients as well as the clinical and methodological heterogeneity and often lack of reporting of clinical features prompting LP across included studies. We therefore used the Joanna Briggs Institute (JBI) case series critical appraisal tool [29]. While this tool does not provide overall ratings or quality thresholds for individual studies, it allowed us to objectively assess individual study quality as it relates to study risk of bias, adequate reporting, and statistical analyses [29]. Two reviewers (DG and JB) performed quality assessments independently and in duplicate. Disagreements were resolved with consensus.

We used the Grading of Recommendations Assessment, Development and Evaluation (GRADE) framework to describe certainty of evidence for the estimate of the incidence of nosocomial meningitis. Using this framework we described concerns related to included study ROB, inconsistency, indirectness, and imprecision (Table II: Diagnostic Utility of Lumbar Puncture in Nosocomial Meningitis). We also performed GRADE assessment on outcomes that were not amenable to pooling (SUPPLEMENTARY TABLE: GRADE ASSESSMENT OF LUMBAR PUNCTURE COMPLICATIONS, RISK FACTORS FOR NOSOCOMIAL MENINGITIS, AND CLINICAL FEATURES ASSOCIATED WITH NOSOCOMIAL MENINGITIS).

**Table II:** Diagnostic utility of lumbar puncture in nosocomial meningitis

Certainty assessment							Impact	Certainty	Importance
№ of studies	Study design	Risk of bias	Inconsistency	Indirectness	Imprecision	Other considerations			
**Incidence of nosocomial meningitis**									
6	Case series	Serious^a^,^b^,^c^	Serious^d^	Serious^e^	Very serious^f^	None		⨁◯◯◯ Very low	CRITICAL

a No study reported the mean duration of hospital stay prior to lumbar puncture.

b Two studies [16,17] did not report the proportion of patients receiving antibiotics prior to lumbar puncture.

c In one study [30] positive CSF culture was required for meningitis, however 75% of patients received antibiotics prior to lumbar puncture.

d There was significant variation in the number of cases of nosocomial meningitis between two studies [31,32] and the other included studies [[15], [16], [17],^30^].

e Variable criteria for nosocomial acquisition was used including two studies that did not provide definition of a nosocomial case [17,30], and one that included individuals who developed symptoms up to one week after hospital discharge [32]. Only one study reported on initial indication for hospital admission [15], and studies provided variable meningitis diagnostic criteria (including not being defined in one study [17]).

f Small number of included participants across studies.

## Results

**Figure 1:**
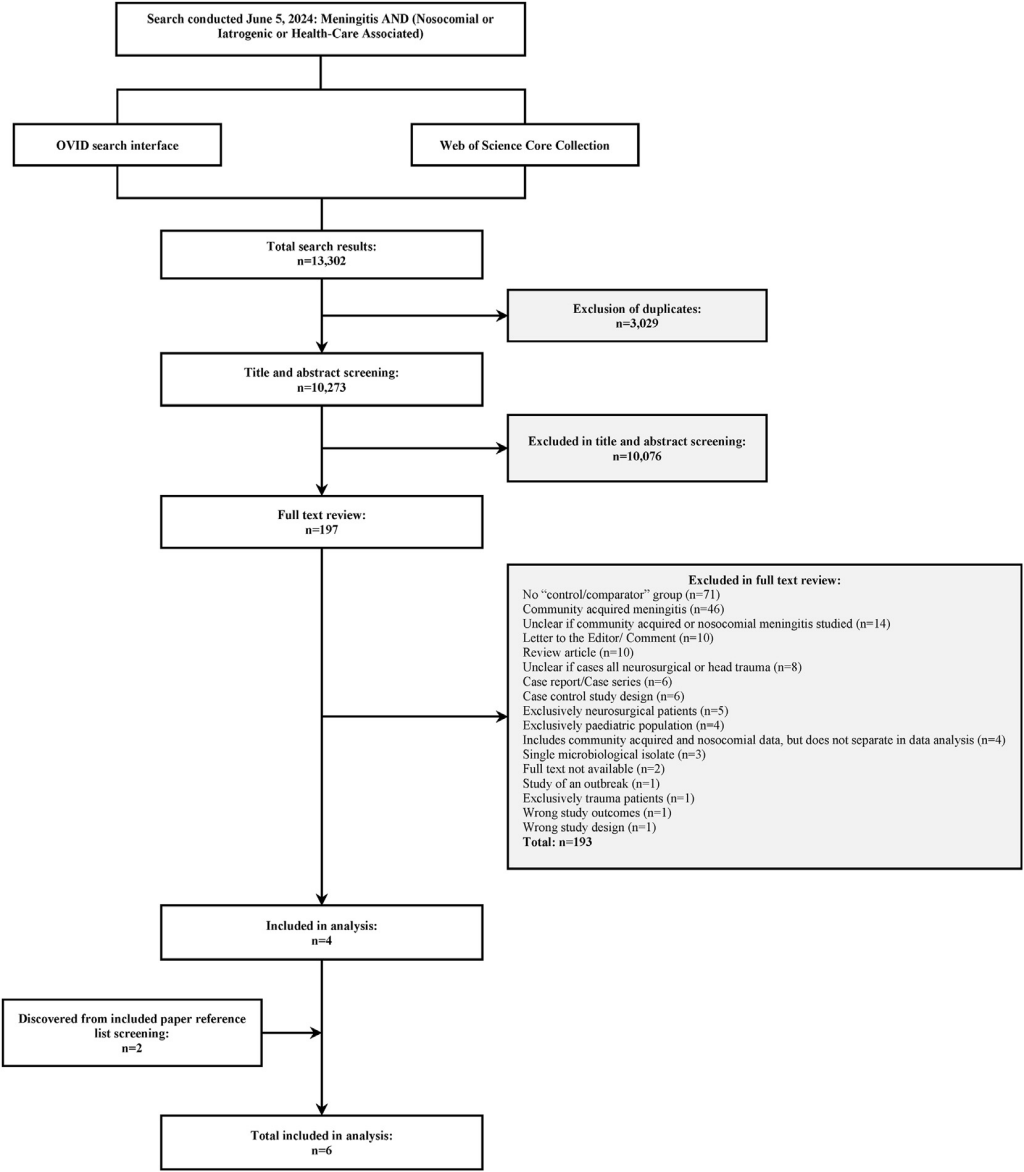
Study identification.

Of 13,302 citations, we reviewed 197 full texts and included six studies (n = 341 patients) in this review (Figure 1: Study Identification) [[15], [16], [17],^30^,^31^]. All included studies were case series. Thirteen non-English studies were screened at the full text stage, none of which met inclusion criteria. Characteristics of included studies are summarized in Table I. Individual study quality assessments using the JBI case series tool are summarized in the supplement (Supplementary Material: JBI Critical Appraisal Tool) [29]. All studies were case series and enrolled between 24 to 114 patients [[15], [16], [17],[30], [31], [32]].

### Study characteristics

In three studies the minimum duration of hospital length of stay prior to LP was at least 48 hours [15,16,31], and in two it was not reported [17,30]. One study mandated a minimum duration of hospital stay prior to LP of 48 hours, but also included individuals who developed symptoms prompting an LP within one week after hospital discharge [32]. Four studies reported the proportion of patients receiving antimicrobials prior to LP, and all were greater than 70% [15,[30], [31], [32]] (although this represented both community and nosocomial acquired cases in one study [31]). Patients were admitted exclusively to an ICU in three studies [15,30,31], two studies included patients in both the ICU and ward setting [17,32], and one did not specify patient location at the time of LP [16]. Definitions of nosocomial acquisition were variable. Two studies defined nosocomial meningitis as a case that occurred greater than 48 hours after admission and using the 1988 Centre for Disease Control and Prevention guidelines [15,31,33], another defined as those in which the indication for LP arose beyond 48 hours of admission [16]. One study defined nosocomial acquisition as cases in which neurological infection was suspected greater than 48 hours after admission or within 1 week of discharge from a previous hospitalization [32]. Definition of nosocomial acquisition was not provided in two studies [17,30]. Meningitis diagnostic criteria were also highly variable among studies and are summarized in Table I. Two studies required positive CSF cultures [16,30] and two required CSF pleocytosis or positive culture [15,31]. One study required isolation of a culprit microbe in CSF or blood along with CSF pleocytosis, but also allowed neurologic infections to be diagnosed “based on the investigator's classification” in the absence of microbiologic confirmation [32]. One study did not define meningitis diagnostic criteria [17].

A GRADE assessment for LP related complications, risk factors for meningitis and diagnostic utility of clinical features in nosocomial meningitis was performed (SUPPLEMENTARY TABLE: GRADE ASSESSMENT OF LUMBAR PUNCTURE COMPLICATIONS, RISK FACTORS FOR NOSOCOMIAL MENINGITIS, AND CLINICAL FEATURES ASSOCIATED WITH NOSOCOMIAL MENINGITIS). We judged each of these outcomes to be at very low certainty of evidence. Therefore, the incidence of LP related complications, risk factors for nosocomial meningitis, and utility of clinical features in diagnosing nosocomial meningitis in this population is uncertain.

### Incidence of nosocomial meningitis

Table II describes the incidence of nosocomial meningitis including the certainty of evidence, and reasons for downgrading certainty. In total, 31 out of 341 (9.1%) of LPs were recorded as positive. One study included two cases of cryptococcal meningitis in patients diagnosed with HIV, one case of tuberculosis meningitis, as well as a patient that received a LP for prolonged obtundation in the setting of known viral meningitis [15]. Additionally, this study also included a patient that received a LP but was already receiving treatment for pneumococcal bacteremia. Another study included two patients with fungal meningitis and one with cerebral toxoplasmosis [32]. It is unclear if these cases represented community acquired or nosocomial meningitis. Excluding these patients, 23 out of 333 (6.9%, very low certainty) of patients had a positive LP, all from two studies [31,32].

## Discussion

This systematic review identified six studies that evaluated the utility of LP to rule out nosocomial meningitis in hospitalized patients who were non-neurosurgical and without head trauma. Despite focusing on only patients where there was sufficient pre-test suspicion of meningitis to prompt an LP, the incidence of nosocomial meningitis was low. There was 31 of 341 (9.1%) positive LPs. After removal of positive LPs that we felt were unlikely to represent nosocomial meningitis from two studies [15,32], the incidence was even lower at 23 of 333 (6.9%) positive LPs. All of these positive LPs were accounted for by two studies [31,32]. It is unclear why there was a higher burden of nosocomial meningitis seen in these studies. However, one included patients with features of possible neurological infection that developed within one week after preceding hospitalization. It is therefore possible that some of these cases could theoretically be community acquired in etiology, however unfortunately this study lacked granularity to discern which of the confirmed cases represented this population. Given this, and that we focused on a population with sufficient clinical suspicion to prompt an LP, we predict the overall incidence of nosocomial meningitis in this population is likely even lower. However, the small number of included participants and studies, frequent administration of antibiotics prior to LP, and variable/absent definitions of nosocomial acquisition and meningitis diagnostic criteria resulted in overall very low certainty of evidence. This significantly limits interpretability and generalizability of the findings.

Due to a small number of included patients as well as heterogenous and lack of reporting in some studies, we were unable to summarize the diagnostic accuracy of individual clinical features in the diagnosis of nosocomial meningitis. In studies examining community acquired meningitis, the reported diagnostic accuracy of individual clinical signs and symptoms is inconsistent, and often poor across studies [3,13,14,[34], [35], [36], [37], [38]]. However, in general, the combination of clinical features are more useful than considering signs or symptoms in isolation. In a large cohort study evaluating 696 episodes of community acquired meningitis, 95% percent of episodes featured at least two out of fever, neck stiffness, altered mental status and headache [13]. The initial JAMA Rational Clinical Exam describing the clinical features predictive of community acquired meningitis had similar findings [34].

Among the included studies in this review, three also reported on a proportion of individuals who underwent LP to rule out community acquired meningitis [16,17,31]. The overall incidence of positive LP in these individuals was 65/355 (18.3%), including both bacterial and non-bacterial etiologies. On review of similar cohort studies where LP was performed to rule out community acquired meningitis, either bacterial or non-bacterial, the incidence of positive LP was variable and between 20-39% of participants [36,38,39]. The overall incidence of nosocomial meningitis in individuals who have undergone neurosurgery has been reported between 0.3 to 8.9%, with individuals requiring internal or external ventricular drainage being at particularly high risk [1,[21], [22], [23],^26^,^27^]. Rates of posttraumatic meningitis have similarly been reported at 1.4%, but up to 11% in certain higher risk injuries [1,20]. In post neurosurgical individuals with clinical features suggestive of meningitis, the rate of positive LP has been reported as high as 28–42% [24,25]. As with community acquired meningitis, the diagnostic accuracy of individual clinical features is often poor and can be especially difficult to interpret in individuals who are sedated or who have recently undergone neurosurgery. Additionally, there is overlap in these features between post operative bacterial meningitis and aseptic meningitis seen after neurosurgery [1,24].

While LP is generally a well-tolerated procedure, it is not without risks. Only two studies reported on LP related complications in our review [15,31]. No complications were reported although this is limited by very low certainty evidence. Frequent minor complications of LP include post-dural puncture headache, transient backache, and nerve root irritation [40,41]. A large systematic review estimated the incidence of post-dural puncture headache at 4.2–11%, and nerve root irritation at 9.2–12.5% (in atraumatic vs. conventional needle respectively), with transient backache estimated at approximately 15% (irrespective of needle type used) [40]. There also exists albeit rare but serious complications of LP including spinal hematoma, post-dural puncture meningitis, and cerebral herniation [[40], [41], [42], [43], [44]]. The incidence of cerebral herniation was estimated at 0.1–3% in a large cohort study [44], with the risk of spinal hematoma similarly low at 0.2% in a separate recent cohort study [43]. The incidence of infection is not well defined, but is also likely quite uncommon [41]. Infection additionally appears to predominantly occur in the setting of epidural or spinal anesthesia. In a case series of 179 episodes of post-dural puncture meningitis, only 17/179 (9%) of episodes occurred after diagnostic LP.

Changes in mental status, delirium and fever are common in admitted patients, [[7], [8], [9], [10]] and LP is a frequently performed procedure. It has been estimated that 360,000 LPs are performed annually in US emergency departments alone [45,46]. Another study found that between 2010 and 2018, the rate of LPs performed in the inpatient setting increased by 5.3%, while the volume of LPs performed in the emergency department decreased by 7.4% [47]. However, it's unclear if this reflects an increase in LPs performed to rule out nosocomial meningitis. In addition to subjecting a patient to possible complications as described previously, the decision to undergo LP is associated with increase healthcare cost, healthcare resource utilization, and radiation exposure if unsuccessful at bedside and ultimately performed by radiology. Balancing the likely low incidence of nosocomial meningitis in this population, with the underlying risks associated with LP and healthcare resource considerations, it may be reasonable to reserve LP in this population only in cases of a very high index of suspicion. However, any conclusions are limited by very low certainty evidence.

This systematic review has several strengths. We pre-registered the protocol on PROSPERO and used a comprehensive search strategy along with independent and paired citation screening and data abstraction. We also formally assessed individual study quality using the JBI case series tool and assessed certainty of evidence using GRADE. To our knowledge, this is the first systematic review to address the incidence of nosocomial meningitis and utility of LP in hospitalized non-neurosurgical and non-trauma patients. We excluded studies that reported exclusively on confirmed positive cases of meningitis, which allowed for a more accurate representation of the incidence of nosocomial meningitis and subsequent utility of LP in this population.

This review also has limitations. First, due to a small number of included patients as well as heterogeneous and lack of reporting in some studies, we were unable to pool many of our pre-specified outcomes including LP related compilations, risk factors for nosocomial meningitis, and performance of individual clinical features in predicting nosocomial meningitis. Second, there was important heterogeneity among included studies, especially with definitions of nosocomial acquisition and meningitis diagnostic criteria. Variable and sometimes lack of reporting of meningitis diagnostic criteria, along with variable and incomplete reporting of pre-LP antibiotic administration make results difficult to interpret. This is further compounded by the lack of widely accepted diagnostic criteria for nosocomial meningitis especially in this population. Notably the three studies that featured patients with a “positive LP” did not mandate a positive CSF culture in their diagnostic criteria [15,31,32]. However, given the high proportion of patients who received antibiotics prior to LP in these studies, it may not be an inappropriate stipulation. Additionally, nosocomial acquisition criteria was poorly and variably reported, and only one study described the initial indications for participant admission [15]. This, along with the lack of widely accepted criteria for nosocomial meningitis in this population restricted us to relying on independent study author definitions for nosocomial acquisition and meningitis diagnostic criteria, limiting certainty of evidence for the estimate of the incidence of nosocomial meningitis. This was compounded by only one study specifying if a time threshold existed that allowed participants with a more remote history of neurosurgery or head-trauma to be included (Table I: Characteristics of Included Studies) [32]. Third, we were only able to identify six studies that met inclusion criteria resulting in a small sample size of 333 patients. This heterogeneity and small sample size resulted in very low certainty evidence. Fourth, despite a comprehensive search strategy and screening over 10,000 citations, it is possible some reports would not be captured given we indexed our search with terms including nosocomial, healthcare-associated, or iatrogenic, and some studies may feature cases of nosocomial meningitis combined with reporting of community acquired cases. Furthermore, we excluded case reports and studies featuring a single microbial isolate such as in outbreak reports as they would not provide data to estimate diagnostic accuracy of clinical features or incidence of nosocomial meningitis. However, given the low incidence of meningitis we saw in this population, it is possible that reporting could exist predominantly in case reports given the infrequent nature of this diagnosis, thus potentially limiting the comprehensiveness of this review and excluding reports that may have allowed a narrative description of features of positive cases. Lastly, while one of our pre-specified outcomes included LP related complications, our search strategy was not specific to LP related complications and as such it is possible some citations reporting this outcome may have been not identified. Further studies examining this patient population would be beneficial to help confirm our findings as well as more definitively assess predictive clinical features and risk factors for nosocomial meningitis in hospitalized individuals without preceding neurosurgery or head trauma.

## Conclusions

In hospitalized adults without preceding neurosurgery or head trauma, the incidence of nosocomial meningitis is low, although this is limited by very low certainty evidence. It may be reasonable to reserve lumbar puncture for instances of very high clinical suspicion of meningitis for the workup of clinical decompensation in these patients. However, further studies are needed to confirm these findings and evaluate clinical features that may help identify patients who may be at highest risk of nosocomial meningitis.

## Conflicts of interest

Dr. Fan reports personal fees from ALung Technologies, Baxter, Getinge, Inspira, Vasomune, and Zoll Medical outside the submitted work. Dr. Bogoch reports consulting fees from NHL Players' Association, Global Affairs Canada (Weapons Threat Reduction Program), as well as grant funding from Canadian Institutes for Health Research, all of which are unrelated to the submitted work. None of the other authors declare any competing interests.

## Funding

There was no specific funding or financial support for this study.

## Author contributions

David Granton and Eddy Fan had full access to all the data in the study and take responsibility for the integrity of the data and the accuracy of the data analysis. The corresponding author (Eddy Fan) attests that all listed authors meet authorship criteria and that no others meeting the criteria have been omitted.

**Eddy Fan:** Conceptualization, Methodology, Validation, Formal Analysis, Investigation, Writing – Original Draft, Writing – Review and Editing, Visualization, Supervision, Project Administration. **David Granton:** Methodology, Validation, Formal Analysis, Investigation, Writing – Original Draft, Writing – Review and Editing, Visualization. **Joseph Brown:** Methodology, Validation, Formal Analysis, Investigation, Writing – Original Draft, Writing – Review and Editing, Visualization. **Shannon Fernando:** Methodology, Validation, Formal Analysis, Investigation, Writing – Review and Editing. **Dipayan Chaudhuri:** Methodology, Validation, Formal Analysis, Investigation, Writing – Review and Editing. **Isaac I. Bogoch:** Methodology, Validation, Formal Analysis, Investigation, Writing – Review and Editing. **Christine Soong:** Methodology, Validation, Formal Analysis, Investigation, Writing – Review and Editing. **Marina Englesakis:** Methodology, Validation, Writing – Review and Editing. **Bram Rochwerg:** Methodology, Validation, Formal Analysis, Investigation, Writing – Review and Editing.

## Prior presentations

This work has not been previously published in any format.

## Data sharing

The data that support the findings of this study are available from the corresponding author upon reasonable request.

## Ethical approval

Given the nature of the study no individual patient level data was used and no specific ethical approval was obtained.

## References

[1] van de Beek D., Drake J.M., Tunkel A.R. (2010). Nosocomial Bacterial Meningitis. N Engl J Med.

[2] Weisfelt M., van de Beek D., Spanjaard L., de Gans J. (2007). Nosocomial bacterial meningitis in adults: a prospective series of 50 cases. J Hosp Infect.

[3] Durand M.L., Calderwood S.B., Weber D.J., Miller S.I., Southwick F.S., Caviness V.S. Jr. (1993). Acute bacterial meningitis in adults. A review of 493 episodes. N Engl J Med.

[4] Hodges G.R., Perkins R.L. (1976). Hospital-associated bacterial meningitis. Am J Med Sci.

[5] Daschner F., Nadjem H., Langmaack H., Sandritter W. (1978). Surveillance, prevention and control of hospital-acquired infections. III. Nosocomial infections as cause of death: retrospective analysis of 1000 autopsy reports. Infection.

[6] Pomar V., Benito N., López-Contreras J., Coll P., Gurguí M., Domingo P. (2013). Spontaneous gram-negative bacillary meningitis in adult patients: characteristics and outcome. BMC Infect Dis.

[7] Rieck K.M., Pagali S., Miller D.M. (2020). Delirium in hospitalized older adults. Hosp Pract (1995).

[8] Ely E.W., Shintani A., Truman B., Speroff T., Gordon S.M., Harrell F.E. (2004). Delirium as a predictor of mortality in mechanically ventilated patients in the intensive care unit. JAMA.

[9] Bleck T.P., Smith M.C., Pierre-Louis S.J., Jares J.J., Murray J., Hansen C.A. (1993). Neurologic complications of critical medical illnesses. Crit Care Med.

[10] Vijapura P., Cowart J.B., Kashiwagi D.T., Killebrew S.R., Burton M.C. (2020). Things We Do For No Reason™: Treatment of Infection-Related Fever in Hospitalized Patients. J Hosp Med.

[11] Kaul D.R., Flanders S.A., Beck J.M., Saint S. (2006). Brief report: incidence, etiology, risk factors, and outcome of hospital-acquired fever: a systematic, evidence-based review. J Gen Intern Med.

[12] Tunkel A.R., Hasbun R., Bhimraj A., Byers K., Kaplan S.L., Scheld W.M. (2017). 2017 Infectious Diseases Society of America’s Clinical Practice Guidelines for Healthcare-Associated Ventriculitis and Meningitis. Clin Infect Dis.

[13] van de Beek D., de Gans J., Spanjaard L., Weisfelt M., Reitsma J.B., Vermeulen M. (2004). Clinical Features and Prognostic Factors in Adults with Bacterial Meningitis. N Engl J Med.

[14] Vibha D., Bhatia R., Prasad K., Srivastava M.V., Tripathi M., Singh M.B. (2010). Clinical features and independent prognostic factors for acute bacterial meningitis in adults. Neurocrit Care.

[15] Jackson W.L., Shorr A.F. (2006). The yield of lumbar puncture to exclude nosocomial meningitis as aetiology for mental status changes in the medical intensive care unit. Anaesth Intensive Care.

[16] Metersky M.L., Williams A., Rafanan A.L. (1997). Retrospective analysis: are fever and altered mental status indications for lumbar puncture in a hospitalized patient who has not undergone neurosurgery?. Clin Infect Dis.

[17] Warshaw G., Tanzer F. (1993). The effectiveness of lumbar puncture in the evaluation of delirium and fever in the hospitalized elderly. Arch Fam Med.

[18] Page M.J., McKenzie J.E., Bossuyt P.M., Boutron I., Hoffmann T.C., Mulrow C.D. (2021). The PRISMA 2020 statement: an updated guideline for reporting systematic reviews. Bmj.

[19] Google Translate, https://translate.google.com/; [accessed April 2023].

[20] Baltas I., Tsoulfa S., Sakellariou P., Vogas V., Fylaktakis M., Kondodimou A. (1994). Posttraumatic meningitis: bacteriology, hydrocephalus, and outcome. Neurosurgery.

[21] Chen C., Zhang B., Yu S., Sun F., Ruan Q., Zhang W. (2014). The incidence and risk factors of meningitis after major craniotomy in China: a retrospective cohort study. PLoS One.

[22] Korinek A.M., Golmard J.L., Elcheick A., Bismuth R., van Effenterre R., Coriat P. (2005). Risk factors for neurosurgical site infections after craniotomy: a critical reappraisal of antibiotic prophylaxis on 4,578 patients. Br J Neurosurg.

[23] Kourbeti I.S., Jacobs A.V., Koslow M., Karabetsos D., Holzman R.S. (2007). Risk factors associated with postcraniotomy meningitis. Neurosurgery.

[24] Li Y., Zhang G., Ma R., Du Y., Zhang L., Li F. (2015). The diagnostic value of cerebrospinal fluids procalcitonin and lactate for the differential diagnosis of post-neurosurgical bacterial meningitis and aseptic meningitis. Clin Biochem.

[25] Maskin L.P., Capparelli F., Mora A., Hlavnicka A., Orellana N., Díaz M.F. (2013). Cerebrospinal fluid lactate in post-neurosurgical bacterial meningitis diagnosis. Clin Neurol Neurosurg.

[26] McClelland S., Hall W.A. (2007). Postoperative central nervous system infection: incidence and associated factors in 2111 neurosurgical procedures. Clin Infect Dis.

[27] Reichert M.C., Medeiros E.A., Ferraz F.A. (2002). Hospital-acquired meningitis in patients undergoing craniotomy: incidence, evolution, and risk factors. Am J Infect Control.

[28] Whiting P.F., Rutjes A.W., Westwood M.E., Mallett S., Deeks J.J., Reitsma J.B. (2011). QUADAS-2: a revised tool for the quality assessment of diagnostic accuracy studies. Ann Intern Med.

[29] Munn Z., Barker T.H., Moola S., Tufanaru C., Stern C., McArthur A. (2020). Methodological quality of case series studies: an introduction to the JBI critical appraisal tool. JBI Evidence Synthesis.

[30] Adelson-Mitty J., Fink M.P., Lisbon A. (1997). The value of lumbar puncture in the evaluation of critically ill, non-immunosuppressed, surgical patients: a retrospective analysis of 70 cases. Intensive Care Med.

[31] Khasawneh F.A., Smalligan R.D., Mohamad T.N., Moughrabieh M.K., Soubani A.O. (2011). Lumbar puncture for suspected meningitis after intensive care unit admission is likely to change management. Hosp Pract (1995).

[32] van Zeggeren I.E., Pennartz C.J., Ter Horst L., van de Beek D., Brouwer M.C. (2024). Diagnostic accuracy of clinical and laboratory characteristics in suspected non-surgical nosocomial central nervous system infections. J Hosp Infect.

[33] Garner J.S., Jarvis W.R., Emori T.G., Horan T.C., Hughes J.M. (1988). CDC definitions for nosocomial infections, 1988. Am J Infect Control.

[34] Attia J., Hatala R., Cook D.J., Wong J.G. (1999). The rational clinical examination. Does this adult patient have acute meningitis?. JAMA.

[35] Fellner A., Goldstein L., Lotan I., Keret O., Steiner I. (2020). Meningitis without meningeal irritation signs: What are the alerting clinical markers?. J Neurol Sci.

[36] Nakao J.H., Jafri F.N., Shah K., Newman D.H. (2014). Jolt accentuation of headache and other clinical signs: poor predictors of meningitis in adults. Am J Emerg Med.

[37] Aronin S.I., Peduzzi P., Quagliarello V.J. (1998). Community-acquired bacterial meningitis: risk stratification for adverse clinical outcome and effect of antibiotic timing. Ann Intern Med.

[38] Tamune H., Takeya H., Suzuki W., Tagashira Y., Kuki T., Nakamura M. (2013). Absence of jolt accentuation of headache cannot accurately rule out meningitis in adults. Am J Emerg Med.

[39] Thomas K.E., Hasbun R., Jekel J., Quagliarello V.J. (2002). The diagnostic accuracy of Kernig's sign, Brudzinski's sign, and nuchal rigidity in adults with suspected meningitis. Clin Infect Dis.

[40] Nath S., Koziarz A., Badhiwala J.H., Alhazzani W., Jaeschke R., Sharma S. (2018). Atraumatic versus conventional lumbar puncture needles: a systematic review and meta-analysis. Lancet.

[41] Wright B.L.C., Lai J.T.F., Sinclair A.J. (2012). Cerebrospinal fluid and lumbar puncture: a practical review. J Neurol.

[42] Baer E.T. (2006). Post-dural puncture bacterial meningitis. Anesthesiology.

[43] Bodilsen J., Mariager T., Vestergaard H.H., Christiansen M.H., Kunwald M., Lüttichau H.R. (2020). Association of Lumbar Puncture With Spinal Hematoma in Patients With and Without Coagulopathy. JAMA.

[44] Costerus J.M., Brouwer M.C., Sprengers M.E.S., Roosendaal S.D., van der Ende A., van de Beek D. (2018). Cranial Computed Tomography, Lumbar Puncture, and Clinical Deterioration in Bacterial Meningitis: A Nationwide Cohort Study. Clin Infect Dis.

[45] Short A., Dunneback E., Stephens J.R., Guidici J., Chatterjee A., Finn E. (2023). Safety and predictors of the success of lumbar punctures performed by a medicine procedure service. J Hosp Med.

[46] Vickers A., Donnelly J.P., Moore J.X., Barnum S.R., Schein T.N., Wang H.E. (2018). Epidemiology of lumbar punctures in hospitalized patients in the United States. PLoS One.

[47] Trunz L.M., Gandhi A.V., Karambelkar A.D., Lange S.M., Rao V.M., Flanders A.E. (2021). National Trends in Lumbar Puncture from 2010 to 2018: A Shift Reversal from the Emergency Department to the Hospital Setting for Radiologists and Advanced Practice Providers. AJNR Am J Neuroradiol.

