# Supplementary material for: Nosocomial meningitis diagnostic test characteristics: a systematic review
Source: Infect Prev Pract. 2024 Sep 23;6(4):100402. doi: 10.1016/j.infpip.2024.100402 (PMC11491709; doi:10.1016/j.infpip.2024.100402)
Supplement: Multimedia component 1 [file mmc1.docx]

**Supplementary Material**

1. **Supplementary Methods**
   1. **Statistical Analysis Plan**
   2. **Initial Search Strategy**
   3. **JBI Critical Appraisal Tool**
   4. **GRADE assessment of lumbar puncture complications, risk factors for nosocomial meningitis, and clinical features associated with nosocomial meningitis**

**Supplementary Methods**:

**Statistical Analysis Plan**

We pooled the prevalence of nosocomial meningitis among eligible studies and the prevalence of each complication associated with lumbar puncture, both with associated 95% confidence intervals (CI) using proportional random effects meta-analysis with variance-stabilizing Freeman-Tukey double arcsine transformation. We also pooled all risk factors assessed for an association with nosocomial meningitis in at least two studies and presented as odds ratios (ORs) and 95% CI using random effects models with DerSimonian–Laird method meta-analysis. For categorical variables in the regression model, we assumed the association between the variable and nosocomial meningitis was linear and the associations across categories were independent of each other and calculated the OR and 95% CI for each category using Bucher’s approach and combined ORs using the inverse variance method to produce a single OR for the variable. If more than one adjusted regression model explored risk factors for nosocomial meningitis in a study, we used only the most adjusted model to avoid clustering.

We will use hierarchical logistic regression modeling to pool the sensitivity, specificity, diagnostic ORs, likelihood ratios, and associated 95% CIs for each sign/symptom of nosocomial meningitis independently by the bivariate linear mixed model. The forest plots and hierarchical summary receiver operating characteristic plots will be created using the estimated model parameters. Q statistics and I² will be used to determine statistical heterogeneity for all outcomes. When there are at least 10 studies available for meta-analysis, we will assess the small-study effects by visual assessment of asymmetry of the funnel plot for each outcome, and Egger’s test. We will perform all statistical analyses using Stata, version 14.0 (StataCorp). All comparisons will be 2-tailed, with a threshold p value of 0.05.

We defined five a priori subgroups: immunocompromised vs immunocompetent patients, ICU versus ward patients, high versus low ROB, minimum length of stay prior to LP (unspecified versus greater than 48 hours versus greater than one week) and indication for LP (fever versus altered LOC versus meningeal signs versus multiple indications). Our hypothesis was that nosocomial meningitis would be more common in immunocompromised patients, patients admitted to ICU, studies with high ROB, studies with shorter minimum admission duration to LP and in patients with multiple indications for LP. For subgroup analysis, when there were at least two studies available for each level (subgroup), we tested for interaction using a χ² significance test. To assess effect modification, when at least 5 studies are available, we ran meta-regression with a modification to the variance of the estimated coefficients suggested by Knapp and Hartung.

**Initial Search Strategy**

The following **databases** were searched from inception (shown in parentheses) via the Ovid search interface: Medline (1946), Medline ePubs and In-Process Citations (daily) (1946), Embase (1947), Cochrane Central Register of Controlled Trials (2014), and Cochrane Database of Systematic Reviews (2005). The Web of Science Core Collection (Clarivate Analytics) was also searched (1900). All the databases were searched on the same day, May 24, 2021. The search was updated on March 7, 2023 and again on June 5, 2024.

The searching process followed the **Cochrane Handbook** (Higgins 1) and the **Cochrane Methodological Expectations of Cochrane Intervention Reviews (MECIR)** (Higgins 2) for conducting the search, the **PRISMA guideline** (Moher 3), and **PRISMA-S** (Rethlefsen 6) extension for searches. The **PRESS guideline for peer-reviewing the search strategies** (McGowan 4), drawing upon the PRESS 2015 Guideline Evidence-Based Checklist, was used to avoid potential search errors.

Preliminary searches were conducted, and full text literature was mined for potential keywords and appropriate controlled vocabulary terms (such as Medical Subject Headings for Medline and EMTREE descriptors for Embase). The **Yale MeSH Analyser** (Grossetta 5) was used to facilitate the MeSH and text word analysis.

The search strategy concept blocks were built on the topics of: **Meningitis** AND (**Nosocomial or Iatrogenic or Health-Care Associated)** using both controlled vocabularies and text word searching for each component. Results were limited to human, adults, and conference abstracts were removed at source where possible.

**JBI Critical Appraisal Tool**

| JBI Critical Appraisal Checklist | | | | | | | | | | |
| --- | --- | --- | --- | --- | --- | --- | --- | --- | --- | --- |
| **Study**  **(Author, Year)** | Were there clear criteria for inclusion in the case series? | Was the condition measured in a standard, reliable way for all participants included in the case series? | Were valid methods used for identification of the condition for all participants included in the case series? | Did the case series have consecutive inclusion of participants? | Did the case series have complete inclusion of participants? | Was there clear reporting of the demographics of the participants in the study? | Was there clear reporting of clinical information of the participants? | Were the outcomes or follow up results of cases clearly reported? | Was there clear reporting of the presenting site(s)/clinic(s) demographic information? | Was statistical analysis appropriate? |
| Jackson, 2006 | N | Y | Y | Y | Y | Y | Y | Y | N | Y |
| Metersky, 1997 | Y | Y | Y | Y | Y | Y | Y | Y | Y | Y |
| Warshaw, 1993 | N | U | U | Y | Y | Y | N | N | N | Y |
| Adelson-Mitty, 1997 | Y | Y | Y | Y | Y | Y | N | Y | N | Y |
| Khasawneh, 2011 | N | Y | Y | Y | Y | Y | Y | Y | N | Y |
| van Zeggeren, 2023 | N | U | U | Y | Y | Y | Y | Y | N | Y |

Y: Yes, N: No, U: Unclear, NA: Not Applicable

**GRADE assessment of lumbar puncture complications, risk factors for nosocomial meningitis, and clinical features associated with nosocomial meningitis**

| **Certainty assessment** | | | | | | | **№ of patients** | | **Effect** | | **Certainty** | **Importance** |
| --- | --- | --- | --- | --- | --- | --- | --- | --- | --- | --- | --- | --- |
| **№ of studies** | **Study design** | **Risk of bias** | **Inconsistency** | **Indirectness** | **Imprecision** | **Other considerations** | **[intervention]** | **[comparison]** | **Relative (95% CI)** | **Absolute (95% CI)** |  |  |
| **LP complications** | | | | | | | | | | | | |
| 2 | Case series | serious^a^ | not serious | not serious | extremely serious^b^ | none | Only two of the included studies addressed this outcome [1, 2]. Both studies did not report any LP related complications. The incidence of LP related complications in this population is uncertain. | | | | ⨁◯◯◯ Very low | IMPORTANT |
| **Risk factors for nosocomial meningitis** | | | | | | | | | | | | |
| 2 | Case series | serious^a,c,d^ | not serious | not serious | extremely serious^e^ | none | Only two of the included studies addressed this outcome [1, 3]. Both studies only reported on history of immunocompromising conditions as a risk factor for the development of nosocomial meningitis. The majority of patients in both studies who had a positive LP had a history of an immunocompromising condition. Underlying risk factors for nosocomial meningitis in this population are unclear. | | | | ⨁◯◯◯ Very low | IMPORTANT |
| **Clinical features associated with nosocomial meningitis: headache** | | | | | | | | | | | | |
| 1 | Case series | serious^a,c^ | not serious | not serious | extremely serious^f^ | none | One study reported on the presence of headache in the case series [3]. Headache did not appear to be significantly associated with nosocomial meningitis in this study. The diagnostic accuracy of headache in this population is unclear. | | | | ⨁◯◯◯ Very low | IMPORTANT |
| **Clinical features associated with nosocomial meningitis: fever** | | | | | | | | | | | | |
| 4 | Case series | serious^a,c,g,h^ | not serious | not serious | very serious^i^ | none | Four studies reported on the presence of fever in the case series [3-6]. Fever did not appear to be significantly associated with nosocomial meningitis across studies. The diagnostic accuracy of fever in this population is unclear. | | | | ⨁◯◯◯ Very low | IMPORTANT |
| **Clinical features associated with nosocomial meningitis: altered level of consciousness** | | | | | | | | | | | | |
| 4 | Case series | very serious^a, c,g,j,k^ | not serious | not serious | very serious^l^ | none | Four studies reported on the presence of altered level of consciousness in the case series [1, 3, 5, 6]. Altered level of consciousness did not appear to be associated with nosocomial meningitis across studies. The diagnostic accuracy of altered level of consciousness in this population is unclear. | | | | ⨁◯◯◯ Very low | IMPORTANT |
| **Clinical features associated with nosocomial meningitis: neck stiffness** | | | | | | | | | | | | |
| 1 | Case series | serious^a,c,m^ | not serious | not serious | extremely serious^f^ | none | One study reported on the presence of neck stiffness in the case series [3]. There did not appear to be an association between neck stiffness and nosocomial meningitis. The diagnostic accuracy of neck stiffness in this population is unclear | | | | ⨁◯◯◯ Very low | IMPORTANT |
| **Clinical features associated with nosocomial meningitis: seizure** | | | | | | | | | | | | |
| 2 | Case series | serious^a,c,d,n^ | not serious | not serious | extremely serious^o^ | none | Two studies [3, 4] reported on the presence of seizure in the case series. Seizure did not appear to be significantly associated with nosocomial meningitis across studies. The diagnostic accuracy of seizure in this population is unclear | | | | ⨁◯◯◯ Very low | IMPORTANT |
| **Clinical features associated with nosocomial meningitis: photophobia or phonophobia - not reported** | | | | | | | | | | | | |
| - | - | - | - | - | - | - | No studies reported this outcome | | | | - | IMPORTANT |
| **Clinical features associated with nosocomial meningitis: laboratory abnormality - not reported** | | | | | | | | | | | | |
| - | - | - | - | - | - | - | No studies reported this outcome | | | | - | IMPORTANT |

**LP:** lumbar puncture

**Explanations**

a. No study described the minimum duration of hospital stay prior to LP

b. Only two studies were included [1, 2] featuring only 82 patients

c. The diagnostic criteria for nosocomial meningitis was ambiguous in one study [3]

d. The diagnostic criteria for nosocomial meningitis varied across studies

e. Only two studies were included featuring only 145 patients [1, 3]

f. Only one study was included [3]

g. The diagnostic criteria for nosocomial meningitis varied across studies and was not defined in one study [6]

h. Variable criteria for nosocomial acquisition was used including two studies that did not provide definition of a nosocomial case [4, 6], and one that included individuals who developed symptoms up to one week after hospital discharge [3]

i. Only four studies were included [3-6] featuring 259 patients. Only one study [3] featured patients with a positive LP.

j. Variable criteria for nosocomial acquisition was used including one study that did not provide definition of a nosocomial case [6] and one that included individuals who developed symptoms up to one week after hospital discharge [3]

k. Variable definitions of altered level of consciousness or mental status changes

l. Only four studies were included [1, 3, 5, 6] featuring 220 patients

m. Nosocomial acquisition definition included patients who developed symptoms up to one week after hospital discharge [3]

n. Criteria for nosocomial acquisition was not reported in one study [4] and included patients who developed symptoms up to one week after hospital discharge in another study [3]

o. Only two studies were included [3, 4] featuring 184 patients

**References**

[1] Jackson WL, Jr., Shorr AF. The yield of lumbar puncture to exclude nosocomial meningitis as aetiology for mental status changes in the medical intensive care unit. Anaesth Intensive Care 2006;34(1):21-4. <https://doi.org/10.1177/0310057x0603400104>.

[2] Khasawneh FA, Smalligan RD, Mohamad TN, Moughrabieh MK, Soubani AO. Lumbar puncture for suspected meningitis after intensive care unit admission is likely to change management. Hosp Pract (1995) 2011;39(1):141-5. <https://doi.org/10.3810/hp.2011.02.384>.

[3] van Zeggeren IE, Pennartz CJ, Ter Horst L, van de Beek D, Brouwer MC. Diagnostic accuracy of clinical and laboratory characteristics in suspected non-surgical nosocomial central nervous system infections. J Hosp Infect 2024;145:99-105. <https://doi.org/10.1016/j.jhin.2023.12.015>.

[4] Adelson-Mitty J, Fink MP, Lisbon A. The value of lumbar puncture in the evaluation of critically ill, non-immunosuppressed, surgical patients: a retrospective analysis of 70 cases. Intensive Care Med 1997;23(7):749-52. <https://doi.org/10.1007/s001340050404>.

[5] Metersky ML, Williams A, Rafanan AL. Retrospective analysis: are fever and altered mental status indications for lumbar puncture in a hospitalized patient who has not undergone neurosurgery? Clin Infect Dis 1997;25(2):285-8. <https://doi.org/10.1086/514531>.

[6] Warshaw G, Tanzer F. The effectiveness of lumbar puncture in the evaluation of delirium and fever in the hospitalized elderly. Arch Fam Med 1993;2(3):293-7. <https://doi.org/10.1001/archfami.2.3.293>.
